# Epidemiological features of tuberculosis infection in a rural prefecture of Japan from 2007 to 2018

**DOI:** 10.1038/s41598-022-17608-y

**Published:** 2022-08-05

**Authors:** Yixiao Lu, Guoxi Cai, Yuhang Liu, Fei He, Kiyoshi Aoyagi

**Affiliations:** 1 Department of Public Health, Nagasaki University Graduate School of Biomedical Sciences, Nagasaki, 852-8523 Japan; 2 Public Health and Hygiene Research Department, Nagasaki Prefectural Institute of Environment and Public Health, Nagasaki, 856-0026 Japan; 3 Department of International Health and Medical Anthropology, Institute of Tropical Medicine (NEKKEN), Nagasaki University, Nagasaki, 852-8523 Japan; 4 Department of Epidemiology and Health Statistics, School of Public Health, Fujian Medical University, Fuzhou, 350122 Fujian China; 5 Department of Epidemiology and Health Statistics, Fujian Provincial Key Laboratory of Environment Factors and Cancer, School of Public Health, Fujian Medical University, Fuzhou, 350122 Fujian China

**Keywords:** Infectious diseases, Tuberculosis

## Abstract

This study aimed to investigate the epidemiological features of reported tuberculosis (TB) infections in a western prefecture (Nagasaki Prefecture) from 2007 to 2018, and to identify the high-risk group for TB infection. The characteristics of 12 years of reported TB infections from the Nagasaki Prefectural Informational Center of Infectious Diseases were summarized by median (interquartile range [IQR]) and proportion; the annual TB infections’ notification rate regarding sex/age was calculated accordingly. The diagnosis of TB infection was made according to clinic symptoms and laboratory examination. In total, 4364 TB infections were reported in 2007 and 2018, with a median age (IQR) of 74 (55–84) years. The majority of TB infections were male (52.6%, 2297/4364), > 65 years (65.8%, 2869/4364), and indigenous (98.1%, 4276/4364). Among active TB, 66.9% (1833/2740) had pulmonary TB, and 25.3% (694/2740) were diagnosed as extrapulmonary TB. The highest notification rate of TB infection was observed in the elderly male population (> 85 years). The annual notification rate of TB infections ranged between 19.4/and 34.0/100,000 for 12 years. The notification rates of TB infections were high in older people of both sexes, especially in men aged > 85. Therefore, appropriate interventions and health management are essential for TB control in (and with a focus on) the elderly population.

## Introduction

Tuberculosis (TB) is a communicable disease caused by a bacillus named *Mycobacterium tuberculosis* (*M. tuberculosis*), which generally affects the lungs (pulmonary TB [PTB]), but can also affect other sites (extrapulmonary TB [EPTB])^1^. People who develop active TB are capable of infecting 5–15 other people by transmitting bacteria through close contact over the course of a year. TB remains one of the top ten causes of death worldwide, with an estimated 10 million people becoming infected in 2017 and 1.6 million fatalities^2^. The World Health Organization (WHO) launched the “End TB Strategy,” with the ultimate goal of eliminating TB^3^ so that by 2035 TB incidence will be reduced to less than ten new tuberculosis cases per 100,000 people per year, and a 95% decrease in deaths. Not only are low- and middle-income countries struggling against the high burden of TB; TB infection remains a persistent problem in high-income countries, especially among numerous vulnerable populations.

Older people are more likely to develop active TB^4,5^. The fact that many developed nations have become aging societies may aggravate this burden. Other vulnerable populations, such as homeless individuals and immigrants from high-burden TB countries, may also accelerate TB diffusion^6,7^. Additional precautions should be taken against the various risks of TB exposure, such as increasing multidrug-resistant (MDR) TB^8^, comorbidity with non-communicable diseases (HIV, diabetes mellitus)^9,10^, alcohol, and tobacco abuse^11^. The improvement of access to high-quality TB care is critical in the plan to eradicate TB^12^.

In Japan, a medium-burden TB country, there were 16,789 newly notified TB infections, and the notification rate per 100,000 people was 13.3 for all forms of TB, according to the Japan Tuberculosis Surveillance Center’s “Annual Report 2018”^13^. More than 60% of newly notified TB infections were among people aged 65 years and above. The most significant number of TB infections were among those aged 85 to 89 (71.1 per 100,000 people), followed by people aged 80 to 84 (45.5 per 100,000 people)^13^. TB notification rates in Japan have fallen significantly since 1980. However, the rise in TB infections among the elderly (≥ 65 years old)^14–16^ and in the younger population (≤ 14 years old)^17^, as well as the expansion of the foreign-born population from high-burden TB countries^18^, may have caused the recent stagnant decline in TB infection. Another non-negligible disparity in Japan’s TB distribution is geographic variation. Considerable variation exists between Japan’s eight regions, with the highest notification rate (17.2 per 100,000 people) in the Kinki region, and the lowest notification rate (8.3 per 100,000 people) in the Tohoku region^13^. Epidemiological research is an integral part of the “End TB Strategy”^19^. Therefore, an in-depth analysis of the TB situations at the first administrative level, then moving to a smaller administrative level, could help implement appropriate and sustainable plans to eliminate TB.

This study aimed to investigate the epidemiological features of notified TB infections in Nagasaki Prefecture during the past decade to identify vulnerable populations among local people.

## Methods

### Study setting

Nagasaki Prefecture is in the northwest part of the Kyushu area and consists of four peninsulas centered around Omura Bay (Kitamatsuura, Nishisonogi, Nagasaki, Shimabara) and three remote islands (Goto, Iki, and Tsushima). Nagasaki Prefecture’s total population shrank from 1,465,517 to 1,339,438 inhabitants between 2007 and 2018; approximately 30% of people were elderly (≥ 65 years).

### Data sources

TB is a notifiable disease under the regulation of the Japanese Ministry of Health, Labour and Welfare (MHLW)^20^, and periodic medical examinations are given to persons residing in the region. TB is defined by a combination of clinical symptoms (i.e., cough, sputum, fever, chest pain, dyspnea, bloody sputum, malaise, loss of appetite) and laboratory tests (positive sputum smear examinations for acid-fast bacilli, isolation of *Mycobacterium tuberculosis* complex by culture, detection of *M. tuberculosis* complex by nucleic acid testing, tuberculin skin tests, interferon-γ release assays, chest radiographs, or clinical decision)^20^. All medical institutions and public health centers report all TB infections through the Notice of Notification on Tuberculosis Patients. The four types of notifiable TB infections are defined as followes^21^: (1) TB patient, or active TB, is diagnosed based on the manifestation of clinical characteristics and detection of pathogens and pathogen genes; (2) asymptomatic pathogen holder (APH), including latent tuberculosis infection (LTBI) diagnosed by laboratory examination but no exhibition of clinical features; and for persons under 5 years old, even the presence of the pathogen cannot be confirmed, thus such cases should be notified due to the high probability of infection such as repeated contact within the range of patient’s droplets; (3) pseudo-symptom case, a person with clinical symptoms and a high probability of being a pseudo-tuberculosis case should be notified given examination of the outbreak situation and epidemiological relevance; (4) corpse of TB disease death, is determined by clinical characteristics and test method; and corpse of suspected TB disease death, as a result of examining a corpse with clinical characteristics. The necessary demographic and epidemiological information of TB infections is reported, including age, sex, address, occupation, contacts during illness, and travel history.

### Study design

We extracted data from the information center for infectious diseases of the Nagasaki Prefectural Institute of Environment and Public Health, which is publicly available at (https://www.pref.nagasaki.jp/bunrui/hukushi-hoken/kansensho/kansen-c/). A total of 4364 TB infections were notified in Nagasaki Prefecture from 2007 to 2018. To calculate the notification rate of TB infections, the age-and sex-stratified population data from 2007 to 2018 were extracted as well. The Nagasaki Prefecture population data are available from the official website of the Nagasaki Prefectural Government.

### Statistical analysis

The characteristics of reported TB infections were summarized by the median with interquartile range (IQR) and the number by proportion. The dataset was categorized by sex, and the Chi-square test or Mann–Whitney U test was conducted to evaluate the statistical significance, with the level of significance set at *p* < *0.05*. The annual notification rate of reported TB infections (all four types of TB notification); of active TB (Type 1 of TB notification); and the annual notification rate of male/female TB infections in the different age groups were calculated. All analyses were performed using RStudio software (Version 1.2.5019 © 2009–2019 RStudio, Inc.)^22^. The ethics committee of Nagasaki Prefectural Institute of Environment and Public Health approved all methods used in this study.

### Ethics declarations

This study was approved by the Research Ethics Committees of Nagasaki Prefectural Institute of Environment and Public Health (No. 2021-9-1). This study did not include any human subjects, and the patient records were anonymized and de-identified before the analysis. The study was conducted according to the guidelines of the Declaration of Helsinki. The need of informed consent was waived by Nagasaki Prefectural Institute of Environment and Public Health ethics committee.

## Results

**Figure 1:**
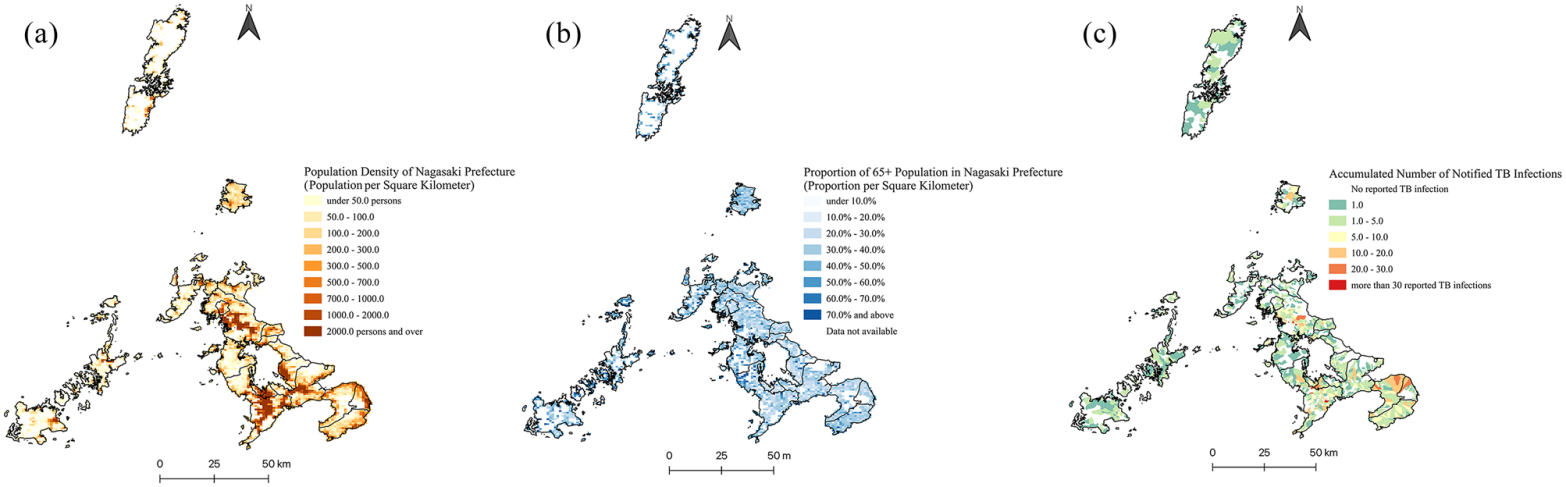
Population distribution and accumulated TB infections from 2007 to 2018 in Nagasaki Prefecture. (**a**) The data of population per 1 km^2^ is from the 2015 population census of Japan; (**b**) The proportion of people older than 65 years per 1 km^2^ was calculated from the 2015 population census of Japan; (**c**) The number of notified TB infections from 2007 to 2018 is accumulated by boundaries of “*machi*” (the 4th level of administrative divisions in Japan).

From 2007 to 2018, 4364 TB infections were reported from 21 districts (13 cities and eight towns) in Nagasaki Prefecture. The population is concentrated in urban areas of Nagasaki city, Sasebo city, Isahaya city, and Shimabara city (Fig. 1a), and rural areas are more strongly affected by population ageing, especially some remote islands like Iki island (Fig. 1b). The reported TB infections were unevenly distributed in Nagasaki Prefecture (Fig. 1c); most cases were reported from densely populated areas; meanwhile, quite a few TB infections were reported from Shimabara peninsula and Iki island.

Table 1 compares the characteristics of reported TB infections by sex, of which 2297 (52.6%) were male. The median age (IQR) of the reported TB infections was 74 (55–84), 65.8% of whom were ≥ 65 years old. Of the reported TB infections, 27.7% (1208) were between 75 and 84 years old, 31.5% (1373) were from Nagasaki City, which has the largest population in the prefecture, and 62.7% (2738) were active TB. According to clinical symptoms, consultation, and epidemiological considerations, droplets/droplet nuclei were identified as the possible transmission routes (3145 infections, 72.1%), and most cases were indigenous (4270 infections, 97.8%). 68.4% of the reported TB infections were unemployed, of which 2620/2983 were people older than 65; among those with a confirmed occupation (1230, 28.2%), 491 (39.9%) reported cases were from the medical and welfare sector.

**Table 1:** Characteristics of 4364 reported TB infections in Nagasaki Prefecture, 2007 to 2018, stratified by sex.

Characteristics	Overall	Male	Female	χ^2^ test/Mann–Whitney U test
	N = 4364	N = 2297 (52.6%)	N = 2067 (47.4%)	*p-value*
**Age, median (IQR)**	74 (55–84)	75 (60–83)	73 (51–84)	*0.03651*
**Age group, n (%)**				< *0.0001*
0–14	55 (1.3)	32 (1.4)	23 (1.1)	
15–24	128 (2.9)	56 (2.4)	72 (3.5)	
25–34	234 (5.4)	96 (4.2)	138 (6.7)	
35–44	289 (6.6)	124 (5.4)	165 (8.0)	
45–54	346 (7.9)	134 (5.8)	212 (10.3)	
55–64	443 (10.2)	248 (10.8)	195 (9.4)	
65–74	690 (15.8)	431 (18.8)	259 (12.5)	
75–84	1208 (27.7)	712 (31.0)	496 (24.0)	
≥ 85	971 (22.3)	464 (20.2)	507 (24.5)	
**Occupation, n (%)**				< *0.0001*
Infant	29 (0.7)	17 (0.7)	12 (0.6)	
Student	87 (2.0)	50 (2.2)	37 (1.8)	
Unemployed	2983 (68.4)	1612 (70.2)	1352 (65.4)	
*(*≥ *65 years, possibly retired)*	*2620 (87.8)*	*1418 (88.0)*	*1156 (85.5)*	
Unknow	363 (8.3)	179 (7.8)	184 (8.9)	
People with a connfirmed occupation, n (%)	1230 (28.2)	439 (19.2)	482 (23.3)	
*Agriculture, forestry*	*67 (5.4)*	*56 (12.8)*	*11 (2.3)*	
*Fishing industry*	*20 (1.6)*	*15 (3.4)*	*5 (1.0)*	
*Construction industry*	*47 (3.8)*	*46 (10.5)*	*1 (0.2)*	
*Manufacturing industry*	*45 (3.7)*	*38 (8.7)*	*7 (1.5)*	
*Electricity, gas, heat supply, water services*	*16 (1.3)*	*16 (3.6)*	*0*	
*Information and communication industry*	*6 (0.5)*	*3 (0.7)*	*3 (0.6)*	
*Transportation industry, postal industry*	*43 (3.5)*	*38 (8.7)*	*5 (1.0)*	
*Wholesale business, retail business*	*79 (6.4)*	*57 (13.0)*	*22 (4.6)*	
*Financial industry, insurance industry*	*4 (0.3)*	*1 (0.2)*	*3 (0.6)*	
*Real estate business, goods leasing business*	*5 (0.4)*	*4 (0.9)*	*1 (0.2)*	
*Academic research, specialized / technical service industry*	*38 (3.1)*	*19 (4.3)*	*19 (3.9)*	
*Accommodation business, restaurant service business*	*53 (4.3)*	*24 (5.5)*	*29 (6.0)*	
*Life-related service industry, entertainment industry*	*14 (1.1)*	*10 (2.3)*	*4 (0.8)*	
*Education and learning support business*	*22 (1.8)*	*12 (2.7)*	*10 (2.1)*	
*Medical and welfare*	*491 (39.9)*	*107 (24.4)*	*384 (79.7)*	
*Service industry (things that are not classified elsewhere)*	*61 (5.0)*	*30 (6.8)*	*31 (6.4)*	
*Public affairs (excluding those classified elsewhere)*	*72 (5.9)*	*37 (8.4)*	*35 (7.3)*	
*Unclassifiable industry*	*147 (12.0)*	*71 (16.2)*	*76 (15.8)*	
**Geographical location, n (%)**				*0.08421*
Nagasaki city	1373 (31.5)	744 (32.4)	629 (30.4)	
Mainland districts	2666 (61.1)	1399 (60.9)	1267 (61.3)	
Isolated islands	325 (7.4)	154 (6.7)	171 (8.3)	
**Notification type, n (%)**		< *0.0001*		
Active TB	2738 (62.7)	1533 (66.7)	1205 (58.3)	
APH*	1585 (36.3)	738 (32.1)	847 (41.0)	
Pseudo-symptom case	35 (0.8)	22 (1.0)	13 (0.6)	
Dead	6 (0.1)	4 (0.2)	2 (0.1)	
**Transmission method, assumption, n (%)**				*0.144*
Droplets/droplet nuclei	3145 (72.1)	1627 (70.8)	1518 (73.4)	
Other	1182 (27.1)	651 (28.3)	531 (25.7)	
Unknown	37 (0.8)	19 (0.8)	18 (0.9)	
**Infected location, assumption, n (%)**				*0.5805*
Domestic	4270 (97.8)	2252 (98.0)	2018 (97.6)	
Abroad	79 (1.8)	37 (1.6)	42 (2.0)	
Unknown	15 (0.3)	8 (0.3)	7 (0.3)	

APH*: asymptomatic pathogen holder (LTBI + persons under observation).

The Chi-square (χ^2^) test/Mann–Whitney U test was used to assess differences in reported TB infection according to sex with a *p-value* less than 0.05 is statistically significant.

Table 2 further describes the 2740 active TB cases (2738 active TB and two active PTB confirmed dead cases). Of these active TB, 56.0% (1534) were male, the median age (IQR) was 74 (55–84), and 76.6% (2099) were senior citizens (≥ 65 years). Of the 2740 active TB, 66.9% (1833) had pulmonary tuberculosis, 25.3% (694) were diagnosed as extrapulmonary tuberculosis, and 7.8% (213) had both. Of the 1833 PTB cases, 62.7% (1149) were smear-positive PTB. Five Drug-Resistant TB (DR TB) cases were notified for the study period; the characteristics of DR TB and its close contact cases are in Supplementary material 1; no Multidrug-Resistant TB or Extensively Drug-resistant TB was reported from 2007 to 2018 in Nagasaki Prefecture.

**Table 2:** Characteristics of 2740 active TB in Nagasaki Prefecture, 2007 to 2018, stratified by sex.

Characteristics	Overall	Male	Female	χ^2^ test/Mann–Whitney U test
	N = 2740^#^	N = 1534 (56.0%)	N = 1206 (44.0%)	*p-value*
Age, Median (IQR)	78 (66–85)	78 (67–84)	79.5 (64–86)	*0.00368*
**Age Group, n (%)**				
0–14	11 (0.4)	5 (0.3)	6 (0.5)	< *0.0001*
15–24	53 (1.9)	24 (1.6)	29 (2.4)	
25–34	86 (3.1)	43 (2.8)	43 (3.6)	
35–44	105 (3.8)	46 (3.0)	59 (4.9)	
45–54	150 (5.5)	73 (4.8)	77 (6.4)	
55–64	236 (8.6)	146 (9.5)	90 (7.5)	
65–74	449 (16.4)	296 (19.3)	153 (12.7)	
75–84	894 (32.6)	528 (34.4)	366 (30.3)	
≥ 85	756 (27.6)	373 (24.3)	383 (31.8)	
**Type of active TB, (%)**				*0.2317*
PTB*	1833 (66.9)	1026 (66.9)	807 (66.9)	
SPPTB*	*1149 (62.7)*	*636 (62.0)*	*513 (63.6)*	
SNPTB*	*684 (37.3)*	*390 (38.0)*	*294 (36.4)*	
EPTB*	694 (25.3)	386 (25.2)	308 (25.5)	
PTB + EPTB	213 (7.8)	122 (8.0)	91 (7.5)	

N = 2740^#^: The 2740 active TB cases described in Table 2 are the sum of 2738 Active TB and 2 Dead (one confirmed of PTB and another of PTB + EPTB) previously mentioned in Table 1;

PTB*: pulmonary tuberculosis; SPPTB*: smear-positive PTB; SNPTB*: smear-negative PTB; EPTB*: extrapulmonary tuberculosis;

The Chi-square (χ^2^) test/Mann–Whitney U test was used to assess differences in active TB according to sex with a *p-value* less than 0.05 is statistically significant.

The reported female TB infections were slightly younger than the males (*p* < *0.05*). 70% of the male TB infections were older than 65, while only 61% of females were older than 65 (*p* < *0.0001*). On the contrary, the females with active TB were slightly older than the males (*p* < *0.05*). 58.7% of the males with active TB were older than 75, while 62.1% of females were older than 75 (*p* < *0.0001*). The majority of reported female TB infections worked in the medical and welfare sector (79.7%), accommodation or restaurant business, and service industry, while the male TB infections primarily worked in medical and welfare sector, wholesale/retail business, agriculture and forestry, and construction industry. The proportion of notified asymptomatic pathogen holders (Type 2 of TB Notification) was significantly higher in females (41.0%) than in males (32.1%). No statistically significance was found regarding the type of active TB between the sexes.

Table 3 portrays the clinical symptoms of the 4364 reported TB infections. The chief pulmonary symptoms include cough, phlegm, fever, chest pain, dyspnea, anorexia, and weight loss. Other symptoms such as night sweats, hemoptysis, erythema nodosum/induratum, and chest X-ray abnormalities (upper lobe infiltrates, cavitation, etc.) strongly suggest a PTB infection^23^. In addition, symptoms of the eight most affected extrapulmonary sites^24^ were stratified by sex and age group. Infections at multiple extrapulmonary sites were observed in both sexes and across all ages, except for 0–14 years; only symptoms of the lymphatic system were noted. Approximately one-quarter of the reported TB infections did not present any TB-related symptoms.

**Table 3:** Clinical symptoms of 4364 reported TB infections by sex and age group, Nagasaki Prefecture, 2007 to 2018.

	n (%)	Pulmonary symptoms		Extrapulmonary involved sites								Asymptomatic	Others
		Main symptoms	Other symptoms	Laryngeal	Pleura	Lymphatic system	Skeletal system	Genitourinary system	Central nervous system	Disseminated	Gastro-intestinal system		
**Sex**													
Male	2297	1398(60.9)	206 (9.0)	6 (0.3)	73 (3.2)	28 (1.2)	27 (1.2)	8 (0.3)	13 (0.6)	10 (0.4)	24 (1.0)	465 (20.2)	39 (1.7)
Female	2067	974 (47.1)	153 (7.4)	5 (0.2)	41 (2.0)	112 (5.4)	38 (1.8)	5 (0.2)	11 (0.5)	11 (0.5)	32 (1.5)	646 (31.3)	39 (1.9)
**Age group**													
0–14	55	6 (10.9)	2 (3.6)	0	0	1 (1.8)	0	0	0	0	0	44 (80)	2 (3.6)
15–24	128	40 (31.3)	9 (7.0)	0	0	6 (4.7)	1 (0.8)	0	0	0	1 (0.8)	66 (51.6)	5 (3.9)
25–34	234	74 (31.6)	18 (7.7)	0	0	7 (3.0)	3 (1.3)	0	1 (0.4)	2 (0.8)	2 (0.8)	124 (53.0)	3 (1.3)
35–44	289	79 (27.3)	13 (4.5)	1 (0.3)	1 (0.3)	11 (3.8)	2 (0.7)	0	1 (0.3)	3 (1.0)	2 (0.7)	171 (59.2)	5 (1.7)
45–54	346	97 (28.0)	26 (7.5)	1 (0.3)	3 (0.9)	13 (3.8)	0	1 (0.3)	3 (0.9)	4 (1.2)	8 (2.3)	183 (52.9)	7 (2.0)
55–64	443	175 (39.5)	34 (7.7)	1 (0.2)	7 (1.6)	23 (5.2)	8 (1.8)	2 (0.5)	4 (0.9)	1 (0.2)	6 (1.4)	175 (39.5)	7 (1.6)
65–74	690	364 (52.8)	64 (9.3)	2 (0.3)	9 (1.3)	25 (3.6)	16 (2.3)	3 (0.4)	4 (0.6)	3 (0.4)	10 (1.4)	173 (25.1)	17 (2.5)
75–84	1208	817 (67.6)	108 (8.9)	3 (0.2)	38 (3.1)	36 (3.0)	25 (2.1)	5 (0.4)	5 (0.4)	7 (0.6)	15 (1.2)	128 (10.6)	21 (1.7)
≥ 85	971	720 (74.2)	85 (8.8)	3 (0.3)	56 (5.8)	18 (1.9)	10 (1.0)	2 (0.2)	6 (0.6)	1 (0.1)	12 (1.2)	47 (4.8)	11 (1.1)

**Figure 2:**
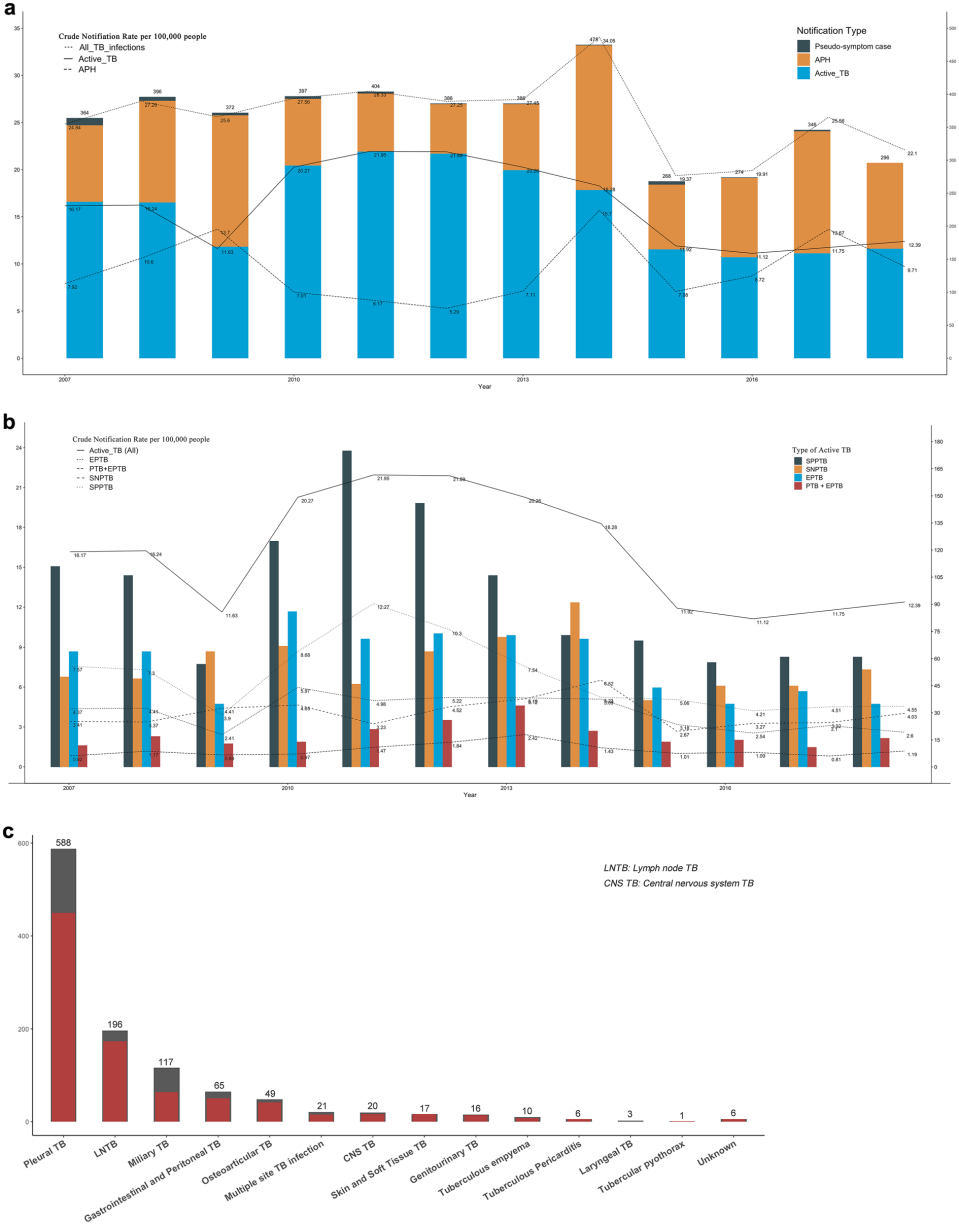
Annual accumulated number and crude notification rate per 100,000 people of TB infections and active TB in Nagasaki Prefecture, 2007 to 2018. (**a**) The notification types of TB infections are active TB, APH (asymptomatic pathogen holder) and pseudo-symptom case, distinguish by colors; (**b**) The types of active TB are SPPTB (smear-positive PTB), SNPTB (smear-negative PTB), EPTB (extrapulmonary TB) and PTB + EPTB, distinguish by colors; (**c**) Accumulated Extrapulmonary Tuberculosis cases in Nagasaki Prefecture, 2007 to 2018. *The black bar graphs represent the number of extrapulmonary TB patients plus those with extrapulmonary TB and pulmonary TB simultaneously; the red bar graphs simply represent the number of extrapulmonary TB patients.

Figure 2a shows the annual accumulated TB infections, the proportion of the three notification types of TB infection (dead not included), the crude notification rate per 100,000 people of TB infections, active TB, and APH from 2007 to 2018.

During the study period, the reported TB infections declined from 2007 (364 in total, with 179 males and 185 females) to 2018 (296 in total, with 156 males and 140 females). The number of accumulated TB infections rose a little from 2007 to 2013, reaching a peak at 475 cases in 2014 (253 males and 222 females), then fell dramatically to 268 in 2015 (143 men and 125 women), and increased slightly in the ensuing years. Among all studying years, the share of asymptomatic pathogen holders (LTBI and persons under observation) decreased between 2009 and 2013, then vastly increased in 2014, and remained stable in the ensuing years; the share of active TB followed an opposite trend. Since 2012, no pseudo-symptom case was notified. The average notification rate of all forms of TB infections per 100,000 people during the study period was 25.8, with a highest at 34.0 in 2014 and lowest at 19.4 in 2015. As for active TB, the average notification rate was 16.2, the highest notification was observed in 2011 (22.0), and the lowest at 9.7 in 2018. The average notification rate of APH was 9.4, the highest notification rate was 15.7 in 2014, and the lowest was 5.3 in 2012.

Figure 2b displays the annual accumulated four active TB types and their crude notification rates, respectively. The smear-positive PTB predominated between 2008 and 2013. After 2013, the notified cases of smear-positive PTB, smear-negative PTB and EPTB tended to distribute equally in the following years. The accumulated number of EPTB is displayed in Fig. 2c. Pleural TB remained the most common form of EPTB, followed by lymph node TB. Most EPTB were older than 65 years (693/865, 80.1%).

**Figure 3:**
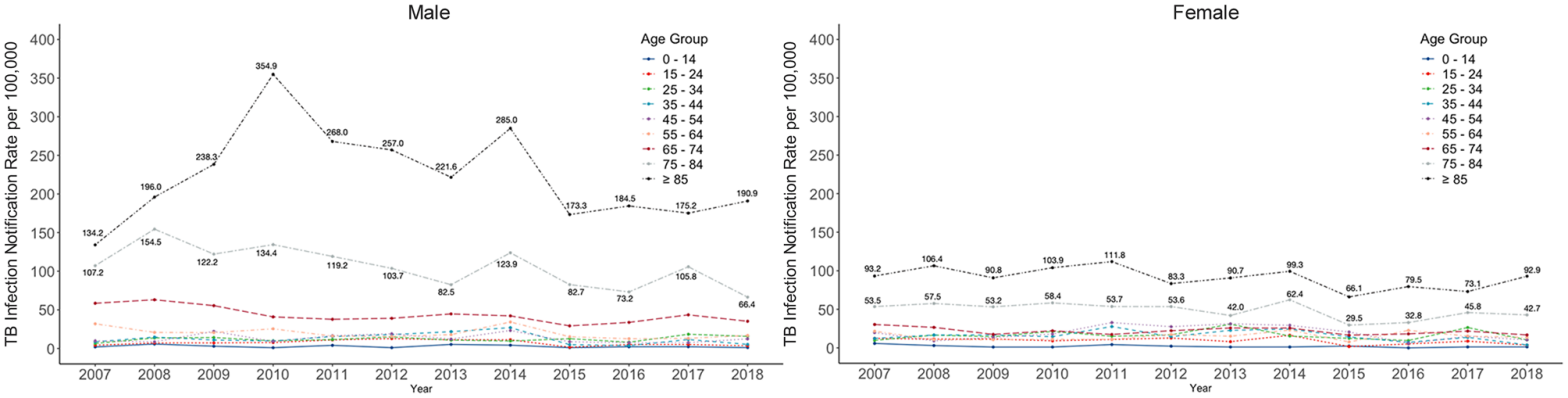
Male/female notification rate in different age groups of TB infections in Nagasaki Prefecture, 2007 to 2018.

Figure 3 shows the sex- and age-stratified notification rate of TB infections per 100,000 people in Nagasaki Prefecture from 2007 to 2018. A much higher notification rate was observed among older people, both men and women, but especially in the elderly male population. Men and women aged > 65 years accounted for 36.8% (1607/4364) and 28.9% (1262/4364) of all TB infections, respectively (Table 1). The highest notification rate was noted in infections aged > 85 years in both sexes, followed by those between 75 and 84 years, and then between 65 and 74 years. A considerable increase in the notification rate was witnessed in men aged > 85 years between 2007 and 2010. The TB infections’ notification rate was relatively stable and low in the youth population and among those ranging in age from 25 to 65 in both sexes. In the age groups of 65–74 years, 75–84 years, and older than 85, the TB infections’ notification rate per 100,000 people in males was two, 2.2, and 2.5 times higher than in females, respectively.

Table 4 summarizes the relevant time intervals of notified TB infections in Nagasaki Prefecture from 2007 to 2018. The median time from onset of symptoms to TB diagnosis for active TB cases was 30 days (IQR: 10–65), and 30 days (IQR: 12–63) for APH cases. Data on the presumed infection date for active TB is available for 503 active TB cases, and the median time from presumed infection date to onset of symptoms was 0 days (IQR: 0–64). The median time from first medical visit to TB diagnosis for active TB cases was four days (IQR: 0–17), and 0 days (IQR: 0–12) for APH cases. Most active TB and APH cases were identified and confirmed within six months.

**Table 4:** Time from presumed infection date to symptom onset, from symptom onset to TB diagnosis, from presumed infection date to TB diagnosis, from the first medical visit to TB diagnosis by active TB and APH, Nagasaki Prefecture, 2007 to 2018.

Time intervals	Active TB (N = 2740)			APH (N = 1585)		
	n	Median (days)	Interquartile range [IQR]	n	Median (days)	Interquartile range [IQR]
From presumed infection date to symptom onset	503	0	0–64	97	19	0–92
From symptom onset to TB diagnosis	1770	30	10–65	461	30	12–63
From presumed infection date to TB diagnosis	667	55	26–148.5	538	118	87.25–159
From first medical visit to TB diagnosis	2740	4	0–17	1585	0	0–12

## Discussion

In this study, we investigated the epidemiological features of reported TB infections in Nagasaki Prefecture from 2007 to 2018. In Japan, the TB notification rate has been falling steadily since 1980^14,15^, and reached a historic low of 13.3 per 100,000 people for all forms of TB in 2017^13^. However, considerable variation exists among Japan’s eight administrative regions. In Nagasaki Prefecture, located in the Kyushu region, the TB burden has remained relatively high over the past decade.

Between 2007 and 2018, there were 4364 reported TB infections in Nagasaki Prefecture, the notification rate of TB infections ranged between 19.4 and 34.0 cases, and the notification rate of active TB varied from 13.5 to 26.9 cases per 100,000 people. A high notification rate of TB infections occurred within the elderly population, especially in men older than 85 years. In the youth population (0–14 years), the TB infections’ notification rate remained below 5 cases per 100,000 people, owing to the nearly 100% coverage rate of the BCG (Bacillus Calmette—Guerin) vaccination from the tuberculosis control program^25^. However, on the other hand, in Nagasaki Prefecture, the high TB infection burden in the elderly population persisted (65.8% of TB infections were aged above 65).

Like other parts of Japan, Nagasaki Prefecture is experiencing a severe population decline and aging transition^26^. Several studies have shown that TB in the elderly population has already become a crucial global health problem^4,27–29^. The susceptibility to respiratory infections, including TB, increases with age, as aging has irreversible effects on both the innate and adaptive immune systems^30,31^. In terms of TB infection pathogenesis, aging affects the processes of the integumental barriers, microbial clearance mechanisms, and cellular immune responses^28,32^. TB in older people is often atypical^33^, or they exhibit non-specific symptoms and signs similar to those of other underlying ailments. Elderly patients may experience more adverse drug reactions, and some prevalent comorbidities in the aging population (e.g., diabetes mellitus) could increase the risk of developing active TB^28^. Although TB in the elderly is mostly due to the reactivation of a former infection^29^, a molecular epidemiological study from Yamagata Prefecture^34^ indicated an association between increased TB infections and the recent transmission of *M. tuberculosis* in elderly people. Further investigation of the TB transmission pattern in Nagasaki Prefecture is required. The proportion of active EPTB patients (25%, 865/2339) in this study was similar to findings from other developed countries and regions: 15% in the U.S.^35^, 22.3% in Hong Kong (China)^36^, and 21.6% in Germany^37^.

Japan first engaged in international efforts to control TB by announcing the “Stop TB Japan Action Plan” in 2008 and initiating the “Stop TB Japan” in 2015, to reduce TB deaths by 10% worldwide^38^. Until 2012, the TB incidence rate was 16.7 per 100,000 people in Japan, which was four to five times higher than that of Western European countries. Japan is still categorized as a medium-burden TB country. Our study also showed that until 2014, the newly notified active TB cases and APH increased slightly in Nagasaki Prefecture. In addition to the high notification rate of TB infections within the elderly population, another notable finding is that the proportion of females working in the medical and welfare sector is very high among notified TB cases within working age. The primary concern regarding TB control is that citizens are losing interest in TB, and the provision of medical services might be degraded due to a lack of TB-related experience among health workers. Also, the gradual replacement of tuberculin skin tests with interferon-γ release assays for LTBI screening makes a higher efficacy diagnosis of TB infection, despite the high rate of BCG vaccination in Japan. After the WHO launched the “End TB Strategy” in 2014^3^, MHLW adopted the WHO’s new strategy by re-addressing priorities and adjusting measures. Extensive screening and treatment of latent TB infection, especially for high-risk groups (the elderly, immigrants, etc.), the restructuring of the medical provision system, and reinforcement of TB control in megacities were considered in this revised strategy. The JATA/Research Institute of Tuberculosis (RIT), the Anti-Tuberculosis Women’s Society, and other related organizations are joining forces to achieve the goal of making Japan a low-burden TB country (with an incidence rate of less than 10 per 100,000 people), as well as a 95% decrease in TB deaths by 2035.

Various factors have contributed to the current TB prevalence in Japan, such as homelessness and MDR-TB issues in the city of Osaka^39^ as well as the presence of immigrants from high TB prevalence countries in Tokyo^40^. Our study shows that in Nagasaki Prefecture, elderly people are a high-risk group for TB infection. Thus, appropriate interventions and management are crucial for TB control in this vulnerable group. Active case findings among senior citizens, the identification of TB transmission settings, strengthening adherence to active TB and LTBI treatment in the older population, training staff in nursing homes, and other feasible interventions are essential to attaining the “End TB Strategy” goal by 2035.

This study has some limitations. First, it is a retrospective study using standard surveillance data from medical institutions and health centers; information on income, living conditions, lifestyle, medical history, and treatment records were not available. Second, the inference regarding the transmission method and the infected location was based only on the conclusions of training physicians. Third, this study does not have data on risk factors for tuberculosis infection, like tobacco use, alcoholism, drug abuse and crowding. Fourth, only a small portion of TB infections in our data was registered with a presumed infection date. Therefore, more completed data are needed to demonstrate the duration of latent infection’s progress to active disease. Finally, the cause of TB infection (the reactivation of latent TB) was unclear.

## Conclusions

This study investigated the epidemiological features of notified TB infections in Nagasaki Prefecture from 2007 to 2018. The overall notification rates of TB infections slightly increased between 2007 and 2014, then decreased in the ensuing years. TB infections mainly were reported in densely populated areas, people aged between 65 to 75 years, and from the medical and welfare sector among notified cases within working age. The notification rates of TB infections were high in older people of both sexes, especially in men aged > 85. Appropriate interventions and health management are urgently needed for TB control in the elderly population.

## Supplementary Information

Supplementary Information.

## Data Availability

The data used in this study were extracted from information center for infectious diseases of the Nagasaki Prefectural Institute of Environment and Public Health, which is publicly available at (https://www.pref.nagasaki.jp/bunrui/hukushi-hoken/kansensho/kansen-c/). The datasets used and/or analysed during the current study are available from the corresponding author on reasonable request.
