# Supplementary material for: Epidemiological features of tuberculosis infection in a rural prefecture of Japan from 2007 to 2018
Source: Sci Rep. 2022 Aug 5;12:13511. doi: 10.1038/s41598-022-17608-y (PMC9355998; doi:10.1038/s41598-022-17608-y)

**Supplementary material 1.** Characteristics of reported Drug-Resistant TB and related close contact cases in Nagasaki Prefecture, 2007 to 2018

ID#: DR-TB patients who are a potential source for TB transmission; ID*: reported TB infections of confirmed close contact with DR-TB patients; INH: isoniazid; RFP: rifampicin; KM: kanamycin; EB: ethambutol; SM: streptomycin


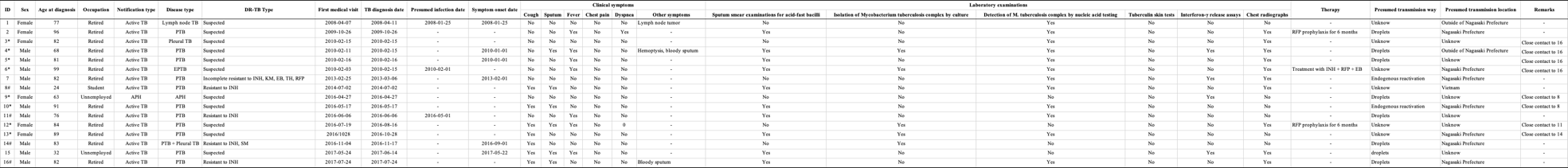

Supplement: Supplementary file 1 — Supplementary Information. [file 41598_2022_17608_MOESM1_ESM.docx]
